# The clinical efficacy and suitable implementation of two extracorporeal blood purification therapies: AN69-oXiris versus PMX-HP

**DOI:** 10.3389/fmed.2024.1344893

**Published:** 2024-01-31

**Authors:** Hye Sung Kim, Yoon Ji Chung, Gyeo Ra Lee, Eun Young Kim

**Affiliations:** Division of Trauma and Surgical Critical Care, Department of Surgery, Seoul St. Mary’s Hospital, College of Medicine, The Catholic University of Korea, Seoul, Republic of Korea

**Keywords:** intra-abdominal septic shock, panperitonitis, extracorporeal blood purification, PMX-HP, AN69-oXiris

## Abstract

**Purpose:**

In septic shock patients, pathogens and excessive endotoxins continuously overstimulate the host’s immune system with a cytokine storm that can lead to multi-organ failure and even mortality. Various types of extracorporeal blood purification treatments have recently been introduced to remove excessive endotoxins and cytokines. Herein, we compared the clinical efficacy of two blood purification methods, PMX-HP and AN69-oXiris, and discussed their detailed indications according to disease severity.

**Materials and methods:**

From December 2016 to April 2023, patients who underwent emergent surgery due to septic shock secondary to peritonitis and subsequently received blood purification treatment with AN69-oXiris or PMX-HP were enrolled. Propensity score (PS)-matching was conducted to adjust for baseline characteristics between the two groups, and the changes in clinical parameters and outcomes were compared. Clinical outcomes were assessed in subgroups of patients who underwent PMX-HP treatment divided according to SOFA scores into low (0–7), intermediate (8–13), and high (> 13) disease severity groups.

**Results:**

Forty patients received blood purification therapy with either PMX-HP or AN69-oXiris during the study period. After 1:2 PS matching, six patients in the AN69-oXiris group and 12 patients in the PMX-HP group were finally analyzed. Vasoactive-inotropic scores (VISs) decreased in both groups after 48 h of treatment compared to the baseline values, but the change in VISs was more pronounced in the PMX-HP group {−57.6 [interquartile range (IQR) = −166.4 – (−10)] vs. -22.9 [IQR = −64–0], respectively, *p* = 0.041}. Decreases in cardiovascular SOFA scores were significantly pronounced in the PMX-HP group [−1.5 (IQR = −4 – 0) vs. 0 (IQR = −1 – 1), respectively, *p* = 0.035]. The 7-day mortality rate was significantly lower than the predicted mortality rate in a subgroup analysis of patients treated with PMX-HP in both the low disease severity group and the intermediate disease severity group.

**Conclusion:**

PMX-HP and AN69-oXiris could be therapeutic options for refractory septic shock patients with intra-abdominal origins, especially after the surgical elimination of the infectious sources. A tailored modality choice that takes into account patient characteristics, such as disease severity and cost burden, could optimize the efficacy of this strategy.

## Introduction

Septic shock is the leading cause of mortality in critically ill patients, with a high mortality rate of 25–30% despite aggressive resuscitation and intensive care of these patients (1–5). One of the major factors contributing to fatal outcomes is the dysregulated immune response during septic shock. When pathogens enter the bloodstream of the host through damaged biological barriers, such as the vascular wall or intestinal mucosa, excessive endotoxin released from the pathogens circulates throughout the body (3, 6). These endotoxin and inflammatory cytokines produced by the host’s immune response could trigger an overwhelming immune response called a cytokine storm (7). If it persists, it causes refractory hypotension and secondary immune paralysis, which can subsequently lead to multi-organ failure and mortality (6). Various types of extracorporeal blood purification treatments have recently been introduced to remove excess cytokines and endotoxins. One type, AN69-oXiris (Gambro Industries, Meyzieu Cedex, France), is a filter that can remove both endotoxin and cytokines and also has a fluid removal function for continuous renal replacement therapy (CRRT) (2, 3). However, concerns have been raised that its endotoxin removal might be insufficient for patients with severe septic shock and high endotoxin concentrations. Another option for extracorporeal blood purification treatment, polymyxin B hemoperfusion (PMX-HP, Toraymyxin, Toray Industries, Tokyo, Japan) selectively adsorbs and eliminates endotoxins from the bloodstream (8), and also has a cytokine-clearing effect (9). PMX-HP is an option for salvage treatment for patients with septic shock who have severe endotoxemia, and several studies reported improvements in complication rates and mortality using this method PMX-HP (10–12). However, it has a higher cost burden compared to the other modalities, lacks the fluid removal function of CRRT, and failed to show improvement in mortality in a recently published large-scale trial (13). Additionally, few studies have compared the clinical efficacy of different hemoperfusion modalities. Thus, no standardized indication for different modalities of hemoperfusion treatment (3, 14, 15). Herein, we compared the clinical effectiveness of AN69-oXiris and PMX-HP in patients who underwent emergent surgery due to abdominal septic shock. We also discussed the indications for two different extracorporeal blood purification treatments for patients with septic shock according to disease severity.

## Materials and methods

### Study design and participants

Since our hospital started PMX-HP treatment in December 2016 and AN69-oXiris treatment in October 2022, we have consistantly conducted blood purification treatment according to each treatment protocol. For the study, patients admitted to the surgical intensive care unit after emergency surgery for septic shock due to peritonitis between December 2016 and April 2023 were eligible for enrollment. Among them, patients who underwent blood purification treatment with AN69-oXiris (AN69-oXiris group) or PMX-HP (PMX-HP group), in addition to standard medical therapy after successful source control, were enrolled. After enrollment, patient data were retrospectively reviewed and analyzed. Propensity score (PS)-matching was conducted to adjust for confounding baseline characteristics and to minimize selection bias due to differences in disease severity between the two groups. Finally, the patients selected after PS-matching were analyzed.

In our institution, PMX-HP treatment, in addition to standard medical therapy after successful source control, has been conducted since 2016 for selected patients diagnosed with septic shock accompanied by an intra-abdominal infection. Successful source control was described by Solomkin et al. as a case in which the following outcomes are attained after surgery or intervention: improvement in fever (oral temperature < 37.5°C), resolution of leukocytosis (white blood cell (WBC) count <12,000 μL/L), improvement in physical findings of tenderness and rigidity, regaining of enteric function, and no requirement for surgery or other intervention (16). The indications for PMX-HP were as follows: (1) age > 18 years; (2) clinical manifestations of sepsis or septic shock in the abdominal cavity with a Sequential Organ Failure Assessment (SOFA) Score > 2; (3) persistence or exacerbation of septic shock despite proper antibiotic treatment and effective source control; and (4) need for high-dose vasopressors within 12 h after diagnosis.

AN69-oXiris has been available at our institution since October 2022, and intensivists have selected one of two modality options, AN69-oXiris or PMX-HP, depending on disease severity as assessed by the initial SOFA scores and inotropic dosage. PMX-HP was preferred when three or more kinds of inotropes were used or when the SOFA score was 10 or above. AN69-oXiris was preferentially considered when using only one type of inotrope, the SOFA score was 5 or less, or CRRT was essential. It was selectively applied in other patients according to the intensivist’s decision. Therefore, the participants in the current study were categorized into the AN69-oXiris group or the PMX-HP group based on the type of filter used. If the patient had any one of the following contraindications for blood purification treatment, neither AN69-oXiris nor PMX-HP were applied: (1) age < 18 years, (2) prior history of PMX-HP hypersensitivity, (3) source control failure, (4) uncontrolled active bleeding, (5) severe leukocytopenia (WBC count <500 μL/L), (6) severe thrombocytopenia (platelet (PLT) count <30,000 × 109/L), (7) hematological malignancy, (8) immunosuppression, and (9) pregnancy. Patients who previously consented to a “do not resuscitate” order or limitations on further treatment, such as renal replacement therapy, were also excluded from this treatment.

All of the patients were diagnosed with septic shock, and the cause of septic shock was suspected to be an intra-abdominal infection based on initial imaging findings or prior culture results. A gram-negative bacteria (GNB) infection was suspected or confirmed based on the source of infection or previous culture results. The diagnosis of sepsis or septic shock was made according to Sepsis-3 guidelines (17). Sepsis was defined as life-threatening organ dysfunction caused by a dysregulated host response to an infection. Septic shock was described as a subset of sepsis requiring a vasopressor to maintain a mean arterial pressure (MAP) of >65 mmHg and a serum lactate level of >2 mmol/L despite adequate volume resuscitation. All patients were treated according to the Surviving Sepsis Campaign (SSC) Guidelines immediately after the diagnosis of sepsis, and the newly updated 2018 SSC guidelines were followed after 2018 (18, 19). As we had already applied the major amendments of the revised 2018 SSC guidelines, such as the application of antibiotics within 1 hour and the earlier use of vasopressors, treatment strategies did not significantly differ by time period. For persisting hypotension due to septic shock, 30 mL/kg of crystalloid solution was administered as fluid resuscitation, and a vasopressor was used if the MAP did not remain above 65 mmHg after. A culture study was performed before administering antibiotics, and empirical antibiotics for intra-abdominal infection were administered immediately thereafter. Antibiotics were adjusted according to the type of bacteria and antibiotic susceptibility after confirming the culture study results in consultation with an infection specialist. Imaging studies such as computed tomography scans and sonography were performed concurrently with initial resuscitation to identify the source of infection. Based on the findings, infectious source control was done as soon as possible through surgical, endoscopic, or radiologic intervention.

### Blood purification treatment protocol

#### A. AN69-oXiris

**Figure 1:**
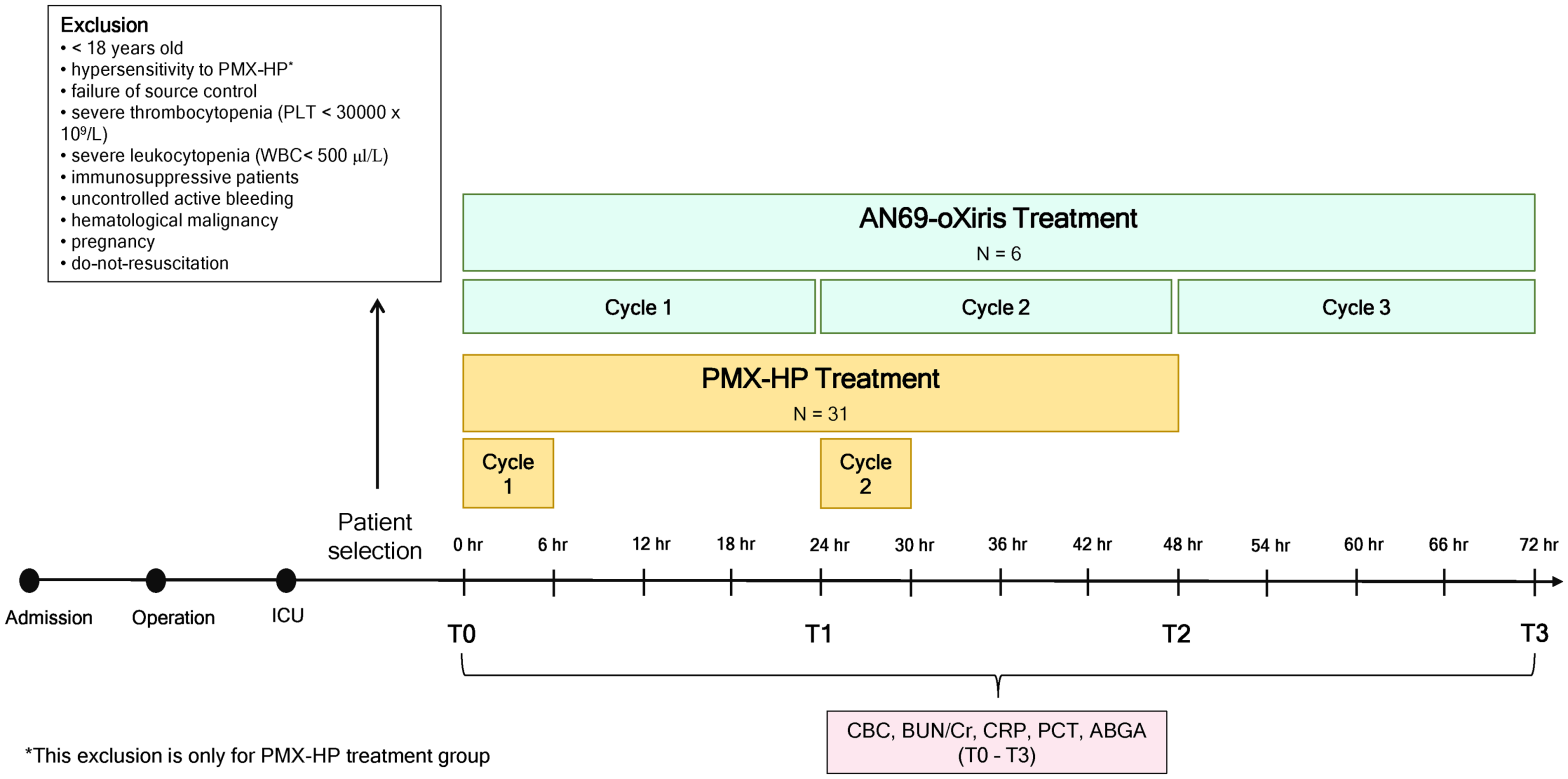
Treatment protocol.

The AN69-oXiris treatment protocol is summarized in Figure 1. The first AN69-oXiris session was initiated within 12 h following surgical source control. A dual-lumen catheter (12Fr Arrow International, Reading, PA, United States) was inserted into the internal jugular vein or femoral vein guided by ultrasound. Subsequently, three consecutive sessions were conducted using the AN69-oXiris hemofilter in a Prismaflex CRRT system (GAMBRO Healthcare, Little Falls, NJ, United States). Each filter was used for 24 h per session. The blood flow rate varied from 100 to 150 mL/min, the replacement rate was set at 150 to 900 mL/h, and the dialysis flow was 700 to 1,200 mL/h. Nafamostat mesylate (Futhan, Torii Pharmaceuticals, Tokyo, Japan) was used as the anticoagulant.

#### B. PMX-HP

The PMX-HP treatment protocol is also summarized in Figure 1. We conducted PMX-HP treatment for patients who decided to undergo PMX-HP according to our guidelines based on the Early Use of Polymyxin B Hemoperfusion in Abdominal Septic Shock (EUPHAS) 1 trial (11). As described in our previous report (20), the first PMX-HP session was initiated within 12 h following surgical source control, and the second PMX-HP session began within 24 h after completion of the first session. Vascular access was accomplished in the same manner using the same catheter as in the AN69-oXiris group. Thereafter, two sessions of PMX-HP were conducted using a toraymyxin cartridge in the Prismaflex machine. The blood flow rate was set between 80 and 120 mL/min, according to the patient, and nafamostat mesylate was used as the anticoagulant. Each session lasted for 6 h, except in cases when PMX-HP treatment discontinuation was indicated. If AKI of KDIGO classification 2 or higher was diagnosed indicating that the serum creatinine (Cr) level rose more than 2 mg/dL over the baseline value or the urine output was less than 0.5 mL/kg/h, the CRRT was additionally applied on a different machine (21). In this case, the same type of Prismaflex machine used for the PMX-HP was also used for the CRRT, and the filter used for the CRRT was the AN69-ST (Baxter, Deerfield, IL, United States).

### Data collection and study outcomes

Data were prospectively collected for each participant from electronic medical records and operative charts from the time of study enrollment and were retrospectively reviewed. Various laboratory values, SOFA scores, immunological parameters, and hemodynamic profiles were measured and analyzed at baseline (T0), 24 h (T1), 48 h (T2), and 72 h (T3) after treatment. Laboratory values including various inflammatory markers such as procalcitonin, C-reactive protein, and presepsin were recorded. Lactate and pH values were obtained from arterial blood gas analysis results. An initial culture study was conducted prior to the administration of antibiotics, and follow-up culture studies were performed from multiple samples, including the blood, sputum, surgical drain, or urine, every 72 h. Hemodynamic status was estimated according to the MAP value, heart rate, and vasoactive requirements represented by the vasoactive-inotropic score (VIS) and the vasopressor dependency index (VDI). Each index was calculated using the worst value during the observation period. All vasopressor doses are expressed as μg/kg/min, and the vasopressin dose is expressed as units/kg/min. The VIS was calculated using the maximum dosing rates of vasopressors and inotropes during the first 6 hours after ICU admission as [VIS = (dopamine dose x1) + (dobutamine dose x1) + (epinephrine dose x100) + (milrinone dose x10) + (vasopressin dose x10,000) + (norepinephrine dose x100)]. We titrated the vasopressor dose to maintain a MAP of 65 mmHg. The dose–response relationship between MAP and the vasopressor dose was expressed as the VDI and defined as an inotropic score divided by MAP as [inotropic score = (noradrenaline dose x100) + (adrenaline dose x100) + (dopamine dose x1) + (dobutamine dose x1) + (phenylephrine dose x100)]. The degree of organ dysfunction was evaluated using the SOFA score (22). Kidney function recovery after treatment was classified into complete recovery, partial recovery, and dependence on dialysis, as described in a study by Guan et al. (7). Complete recovery was defined as a return to normal serum Cr levels after 90 days of follow-up. Partial recovery was defined as an insufficient return to normal serum Cr levels and/or persistent hematuria and proteinuria after 90 days of follow-up but without the need for hemodialysis. Dependence on dialysis was defined as the need for hemodialysis after 90 days of follow-up.

The occurrence of any postoperative complications was recorded during the study period and graded according to the Clavien-Dindo classification (23). Complications of grade III or more were analyzed. Grade III complications were cases requiring surgical, radiologic, or endoscopic interventions. Grade IV complications were cases showing life-threatening morbidities, and grade V complications were defined as death. We reviewed the occurrence of any death during hospitalization and 7-day and 28-day mortality after surgery. The primary outcome of the current study was changes in various parameters, such as hemodynamic profiles and SOFA scores, during 24 h after the initiation of blood purification treatment using various filters (AN69-oXiris or PMX-hemoperfusion). The secondary outcome was mortality and total cost during the ICU stay or hospitalization. Informed consent was obtained from all participants, and if the participant was unable to express his or her opinion clearly, consent was obtained from their guardian. This study was approved and monitored by the Institutional Review Board of our institution (IRB No. KC23RASI0379) and was conducted according to the Declaration for Helsinki and its later amendments.

### Statistical analysis

All statistical analyses were performed using the SPSS statistical package software for Windows (version 24.0, SPSS Inc., Chicago, IL, United States). Categorical variables are presented as proportions and were analyzed using the χ2 test or Fisher’s exact test. Continuous variables are described as medians [interquartile range (IQR)]. A logistic regression analysis of clinical factors, including age, initial total SOFA score, and SOFA score for each item, the time between ICU admission and the initiation of treatment, the infection site, the prothrombin time, and lactate value, was performed in patients who underwent PMX-HP treatment to estimate the PS. We used 1:2 matching, and a caliper width equal to 0.01 of the standard deviation of the logit of the PS was used. In the PS-matched population, we compared the continuous variables using a paired *t*-test or Wilcoxon rank one-way ANOVA to compare the two groups. A *p*-value of less than 0.05 was considered statistically significant.

## Results

**Figure 2:**
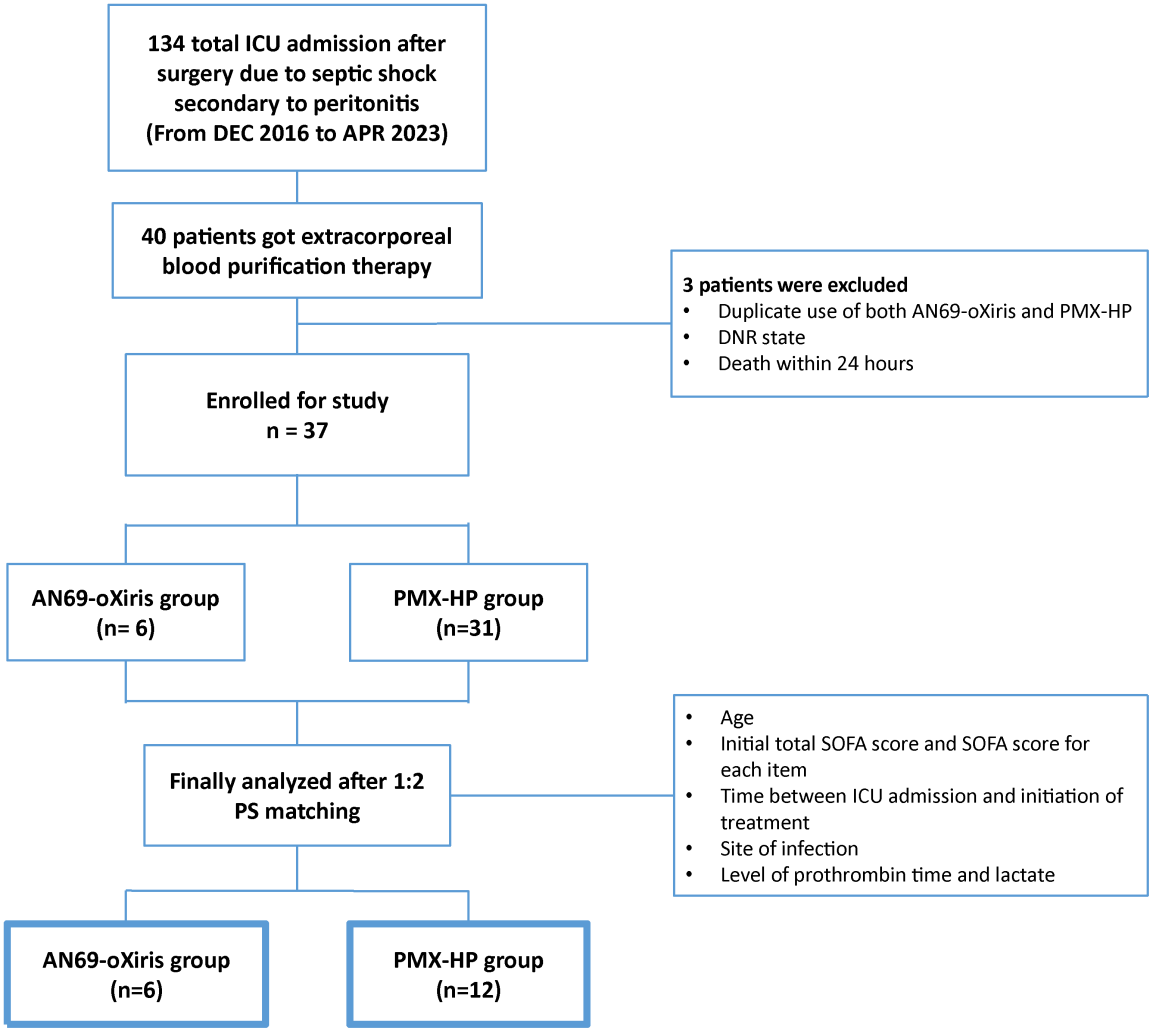
Diagram of patient enrollment.

During the study period, 134 patients were admitted to the surgical ICU after emergent surgery due to septic shock secondary to peritonitis. Among them, a total of 40 patients received blood purification therapy, and three were excluded from study enrollment: one who had both AN69-oXiris and PMX-HP treatment, one who was designated as “do not resuscitate” after blood purification treatment, and another who died within 24 h of treatment initiation. Finally, 37 patients were eligible for enrollment (Figure 2). The demographics and disease profiles of all patients are shown in Table 1. Initially, there were significant differences in coagulative SOFA scores, the time between ICU admission and initiation of treatment, infection sites, prothrombin times, and lactate levels between the two groups. After PS-matching, six patients (33.3%) in the AN69-oXiris group and 12 patients (66.7%) in the PMX-HP group were finally selected, and a comparative analysis was performed. No significant difference in distribution was seen between the two groups in terms of demographics or disease profiles (Table 2). The mean age of the participants was 69 years (IQR = 61–78). The median SOFA score at ICU admission was 8 (IQR = 7–11), and the median time between ICU admission and the initiation of treatment was 5.5 h (IQR = 2–14). 18 out of 31 patients in the PMX-HP group applied CRRT, and the same type of modality of CRRT as CVVHDF was applied. We used nafamostat mesylate in 7 patients for circuit anticoagulation, and 11 patients underwent CRRT treatment without anticoagulation agents. Also, the mean duration of CRRT treatment was 135 h and 9 min, and the mean usage time of one circuit was+23 h and 28 min. In the AN69-oXiris group, all 6 patients had CRRT with the AN69-oXiris filter, and anticoagulation was napamostat mesylate for all patients. For the PMX-HP group, 56 filters were used in 31 patients, and all completed the planned trial time; however, for the AN69-oXiris group, a total of 15 filters were used in 6 patients over the entire study period, of which 12 filters (80%) were used for the planned trial time of 24 h, and 3 filters (20%) were replaced before then due to clogging.

**Table 1:** Demographic and disease profiles of the study group population before PS-matching.

Variables	Total	AN69-oXiris group	PMX-HP group	*p*-value
	*N* = 37	*N* = 6 (16.2%)	*N* = 31 (83.8%)	
Demographic characteristics [Median (range or %)]				
Age, years (median [IQR])	69 (59, 78)	67 (59.8, 75.8)	69 (59, 78)	0.801
Male, *n* (%)	16 (43.2)	2 (33.3)	14 (45.2)	0.680
BMI, kg/m^2^ (median [IQR])	22 (20.3, 23.9)	21.9 (19.4, 21.9)	22 (20.8, 24.4)	0.779
SOFA score at ICU admission (median [IQR])	10 (7.5, 12)	8 (5.8, 10.3)	10 (8, 14)	0.079
Respiratory SOFA	3 (0.5, 3)	1 (0, 3)	3 (2, 3)	0.110
Coagulative SOFA	1 (0, 2)	0 (0, 0.3)	1 (0, 2)	0.003
Liver SOFA	1 (0, 1)	0 (0, 0.5)	0 (0, 1)	0.531
Cardiovascular SOFA	4 (4, 4)	3.5 (0, 4)	4 (4, 4)	0.151
CNS SOFA	1 (0, 2)	1.5 (0, 2.3)	1 (0, 2)	0.892
Renal SOFA	1 (0, 2)	1.5 (0.75, 4)	1 (0, 2)	0.153
APACHE II score at ICU admission (median [IQR])	20 (15.5, 26.5)	12 (10.5, 23.5)	20 (17, 27)	0.069
Time between ICU admission and initiation of treatment, hr (median [IQR])	12 (4, 15)	3 (1, 9)	13 (10, 15)	0.016
Underlying disease, *n* (%)				
Hypertension	19 (51.4)	4 (66.7)	15 (48.4)	0.660
Diabetes	11 (29.7)	3 (50)	8 (25.8)	0.335
CVA	8 (21.6)	1 (16.7)	7 (22.6)	1.000
Heart failure	2 (5.4)	1 (16.7)	1 (3.2)	0.302
Chronic kidney disease without dialysis^a^	5 (13.5)	2 (33.3)	3 (9.7)	0.177
End stage renal disease	4 (10.8)	2 (33.3)	2 (6.5)	0.115
Site of infection, *n* (%)				
Upper GI (1+2)	9 (24.3)	4 (66.7)	5 (16.1)	0.022
Lower GI (3)	25 (67.6)	2 (33.3)	23 (74.2)	0.073
Hepatobiliary (4+5)	1 (2.7)	0	1 (3.2)	1.000
Miscellaneous (9)	2 (5.4)	0	2 (6.5)	1.000
AKI stage at ICU admission (KDIGO), *n* (%)		4 (66.7)	26 (83.9)	0.601
Stage 1	4 (10.8)	1 (16.7)	3 (9.7)	0.524
Stage 2	14 (37.8)	1 (16.7)	13 (41,9)	0.376
Stage 3	12 (32.4)	2 (33.3)	10 (32.3)	1.000
CRRT prescription				
CRRT machine (*n*, %)				
Prismaflex CRRT system	24 (100)	6 (100)	18 (100)	–
CRRT filter (*n*, %)				
AN69-oXiris	6 (25)	6 (100)	0	–
AN69-ST	18 (75)	0	18 (100)	–
CRRT modality (*n*, %)				
CVVHDF	24 (100)	6 (100)	18 (100)	–
Blood flow rate (*n*, %)	148.8 ± 29.5	146.7 ± 8.2	149.4 ± 34.03	0.847
Circuit anticoagulation (Nafamostat mesylate) (*n*, %)	13 (54.2)	6 (100)	7 (38.9)	0.016
Duration of CRRT treatment, h (mean±SD)	128.1 ± 66.8	85.2 ± 40.4	142.4 ± 68.5	0.068
Average circuit duration, h (mean±SD)	25.2 ± 15.4	23.0 ± 10.6	25.8 ± 16.7	0.727
Baseline laboratory findings				
WBC, ×10^9^counts/L (median [IQR])	3980 (2415, 15165)	10245 (1683, 15528)	3790 (2460, 14909)	0.640
Hemoglobin, g/㎗ (median [IQR])	10.1 (8.9, 11)	10.4 (9.3, 11.2)	10 (8.8, 11.1)	0.880
Platelet, ×109counts/L (median [IQR])	150 (93, 196.5)	179 (151, 218.8)	138 (57, 193)	0.410
Prothrombin time (median [IQR])	47.1 (30.8, 61.8)	67.6 (49.4, 97.4)	37.6 (29.8, 58.6)	0.003
BUN, mg/dl (median [IQR])	32.4 (24.7, 39.5)	45.1 (28.4, 81.5)	30.7 (23.4, 36.7)	0.016
Creatinine, mg/dl (median [IQR])	1.81 (1.18, 2.88)	2.37 (1.52, 6.58)	1.61 (1.17, 2.44)	0.118
C-reactive protein, mg/dl (median [IQR])	12.79 (5.79, 24.22)	5.28 (0.61, 23.09)	13.25 (7.2, 25.75)	0.317
Procalcitonin, ng/mL (median [IQR])	67.71 (21.94, 107.81)	30.84 (13.36, 221.88)	73.71 (24.38, 103.09)	0.564
Presepsin, pg/mL (median [IQR])	1044 (649, 2170)	1303 (951, 3353.8)	1035 (625, 1876)	0.949
Lactate, mmol/L (median [IQR])	5 (4.1, 7.7)	3.14 (1.18, 6.1)	5.4 (4.4, 8.1)	0.039
Culture, *n* (%)				
Gram positive bacteria	24 (64.9)	5 (83.3)	19 (61.3)	0.394
Gram negative bacteria	20 (54.1)	4 (66.7)	16 (51.6)	0.667
Fungus	2 (5.4)	0	2 (6.5)	1.000
Not identified	8 (21.6)	0	8 (25.8)	0.305

IQR, interquartile range; CVVHDF, continuous venovenous hemodiafiltration; SD, standard deviation.

a Chronic kidney disease is defined as abnormalities of kidney structure or function, present for>3months, and its stage is classified via eGFR based on KDIGO guideline.

**Table 2:** Demographic and disease profiles of the study group population after PS-matching (1:2).

Variables	Total	AN69-oXiris group	PMX-HP group	*p*-value
	*N* = 37	*N* = 6 (16.2%)	*N* = 31 (83.8%)	
Demographic characteristics [Mean ± SD (range or %)]				
Age, years (median [IQR])	69 (61, 78)	67 (60, 76)	71 (63, 78)	0.608
Male, *n* (%)	6 (33.3)	2 (33.3)	4 (33.3)	1.000
BMI, kg/m^2^ (median [IQR])	22.1 (21.0, 23.2)	21.9 (19.4, 23.6)	22.2 (21.0, 24.3)	0.927
SOFA score at ICU admission (median [IQR])	8 (7, 11)	8 (6, 10)	8 (7, 12)	0.328
Respiratory SOFA	3 (0, 3)	1 (0, 3)	3 (1, 4)	0.180
Coagulative SOFA	1 (0, 0)	1 (0, 0)	1 (0, 0)	1.000
Liver SOFA	1 (0, 0)	1 (0, 1)	1 (0, 0)	0.621
Cardiovascular SOFA	4 (4, 4)	3.5 (0, 4)	4 (4, 4)	0.122
CNS SOFA	1 (0, 2)	1.5 (0, 2)	1 (0, 2)	0.785
Renal SOFA	1 (0, 2)	1.5 (1, 4)	1 (0, 2)	0.239
APACHE II score at ICU admission (median [IQR])	20 (12, 24)	12 (11, 24)	21 (18, 25)	0.168
Time between ICU admission and initiation of treatment, hr (median [IQR])	5.5 (2, 14)	3 (1, 9)	11 (2, 14)	0.251
Underlying disease, *n* (%)				
Hypertension	11 (61.1)	4 (66.7)	7 (58.3)	1.000
Diabetes	7 (38.9)	3 (50)	4 (33.3)	0.627
CVA	6 (33.3)	1 (16.7)	5 (41.7)	0.600
Heart failure	1 (5.6)	1 (16.7)	0	0.333
Chronic kidney disease without dialysis^a^	4 (22.2)	2 (33.3)	2 (16.7)	0.569
End stage renal disease	4 (22.2)	2 (33.3)	2 (16.7)	0.569
Site of infection, *n* (%)				
Upper GI (1+2)	7 (38.9)	4 (66.7)	3 (25)	0.141
Lower GI (3)	11 (61.1)	2 (33)	9 (75)	0.141
Hepatobiliary (4+5)	0	0	0	
Miscellaneous (9)	0	0	0	
AKI stage at ICU admission (KDIGO), *n* (%)	15 (83.3)	4 (66.7)	11 (91.7)	0.245
Stage 1	2 (11.1)	1 (16.7)	1 (8.3)	1.000
Stage 2	5 (27.8)	1 (16.7)	4 (33.3)	0.615
Stage 3	8 (44.4)	2 (33.3)	6 (50)	0.638
Baseline laboratory findings				
WBC, ×10^9^counts/L (median [IQR])	4190 (2333, 14400)	10245 (1683, 15528)	3570 (2393, 10008)	0.378
Hemoglobin, g/㎗ (median [IQR])	10.7 (9.8, 11.3)	10.4 (9.3, 11.2)	10.7 (9.8, 11.3)	0.477
Platelet, ×109counts/L (median [IQR])	178.5 (160, 213)	179 (151, 219)	178.5 (154, 217)	0.581
Prothrombin time (median [IQR])	56.7 (42.0, 73.1)	67.55 (49.4, 97.4)	55.7 (36.0, 68.1)	0.125
BUN, mg/dl (median [IQR])	32.9 (26.7, 53.7)	45.1 (28.4, 81.5)	28.6 (26.2, 40.3)	0.210
Creatinine, mg/dl (median [IQR])	1.82 (1.15, 3.00)	2.37 (1.51, 6.58)	1.65 (1.10, 2.90)	0.392
C-reactive protein, mg/dl (median [IQR])	8.62 (3.02, 16.19)	5.28 (0.61, 23.09)	8.62 (5.05, 14.60)	0.886
Procalcitonin, ng/mL (median [IQR])	50.57 (21.37, 125.30)	30.84 (13.36, 221.88)	82.48 (25.05, 129.82)	0.567
Presepsin, pg/mL (median [IQR])	1068 (639, 2715)	1303 (951, 3354)	832 (564, 3686)	0.788
Lactate, mmol/L (median [IQR])	4.44 (3.18, 5.80)	3.135 (1.18, 6.10)	4.5 (3.58, 6.60)	0.247
Culture, *n* (%)				
Gram positive bacteria	14 (77.8)	5 (83.3)	9 (75)	1.000
Gram negative bacteria	12 (66.7)	4 (66.7)	8 (66.7)	1.000
Fungus	0	0	0	
Not identified	2 (11.1)	0	2 (16.7)	0.529

IQR, interquartile range.

a Chronic kidney disease is defined as abnormalities of kidney structure or function, present for>3months, and its stage is classified via eGFR based on KDIGO guideline.

**Figure 3:**
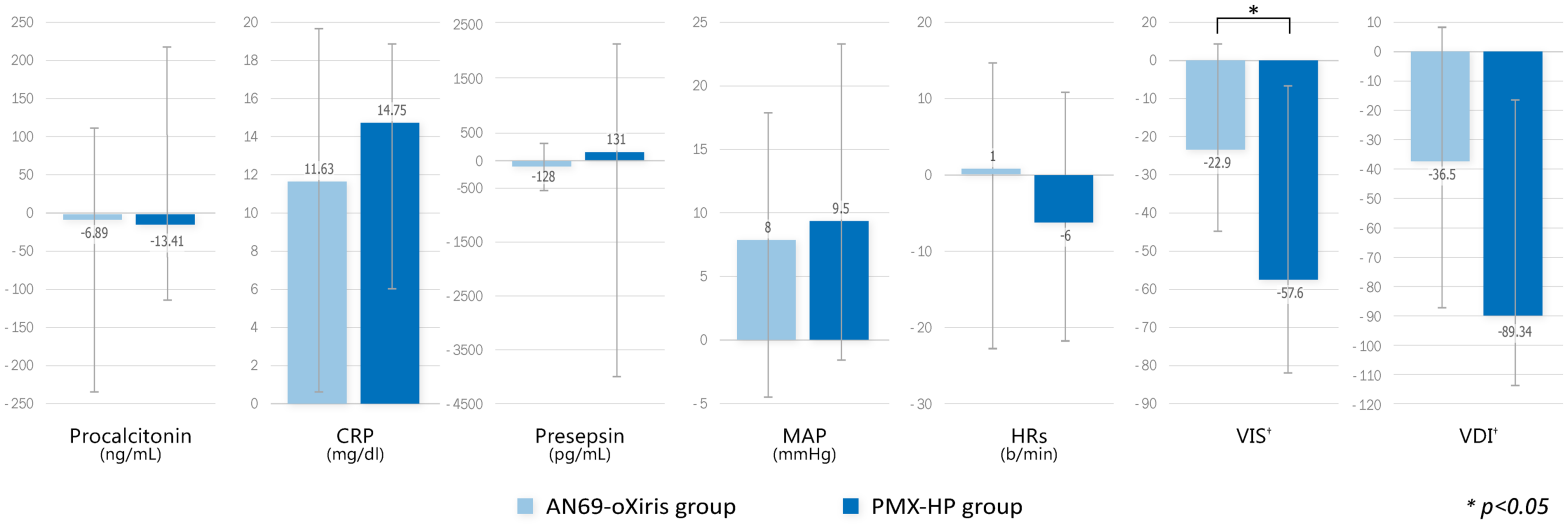
Comparison of changes in immunologic parameters and hemodynamic profiles after blood purification with different filters (modalities). Change of each value = T1 – T0 (^†^Change of VIS, VDI = T2 – T0).

Figure 3 demonstrates the changes in immunologic parameters and hemodynamic profiles after blood purification with each filter. The change in each value represents the difference from baseline to T1, and for VIS and VDI only, the change from baseline to T2. There were no substantial changes in immune markers or hemodynamic profiles in the AN69-oXiris group. However, C-reactive protein (CRP) levels were significantly higher than the baseline values in the PMX-HP group (*p* = 0.005). Regarding hemodynamic profiles, VISs and VDI values in the PMX-HP group decreased significantly after 48 h of treatment compared to baseline (*p* = 0.001 and *p* = 0.003, respectively). Additionally, changes in VISs were more apparent in the PMX-HP group than in the AN69-oXiris group {−57.6 [IQR = −166.4 – (−10)] vs. −22.9 [IQR = −64–0], respectively, *p* = 0.041}. The PMX-HP group was divided into 18 patients who received CRRT and 13 patients who did not, and the change in VIS from baseline to 48 h after treatment was compared and found no significant difference between two groups {−91.3 [IQR = −147.4 – (−62.4)] vs. −45.8 [IQR = −116.7 – (−20)], respectively, *p* = 0.158}.

**Figure 4:**
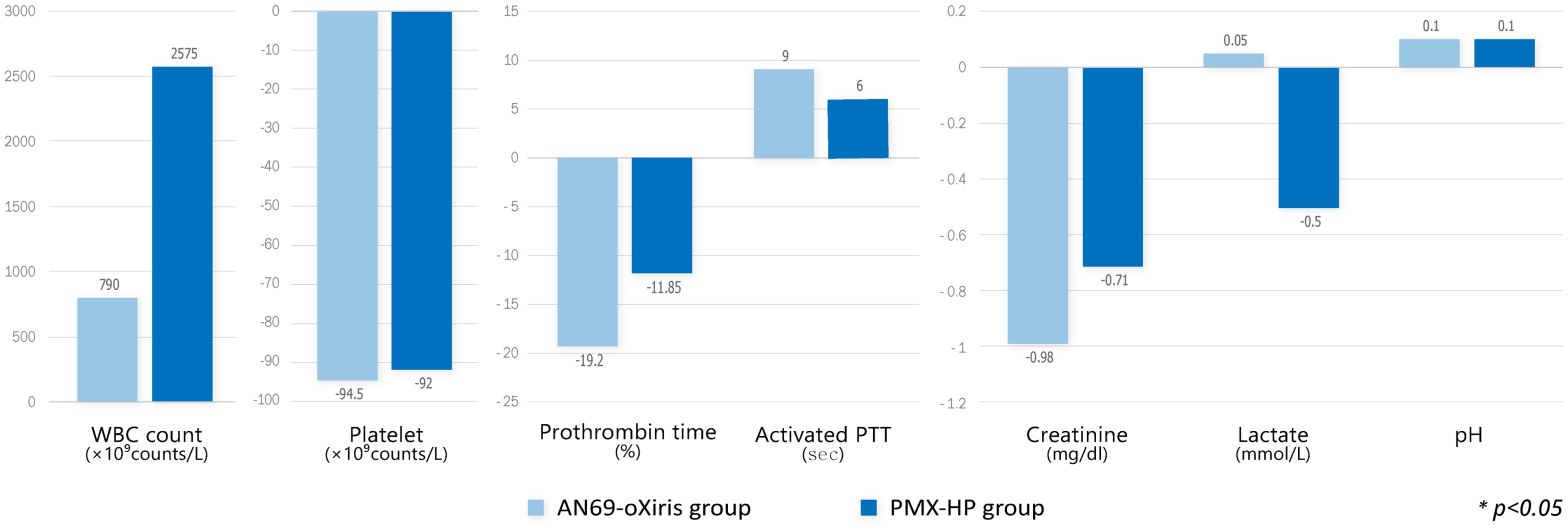
Comparison of changes in laboratory values after blood purification with different filters (modalities). Change of each value = T1 – T0.

**Figure 5:**
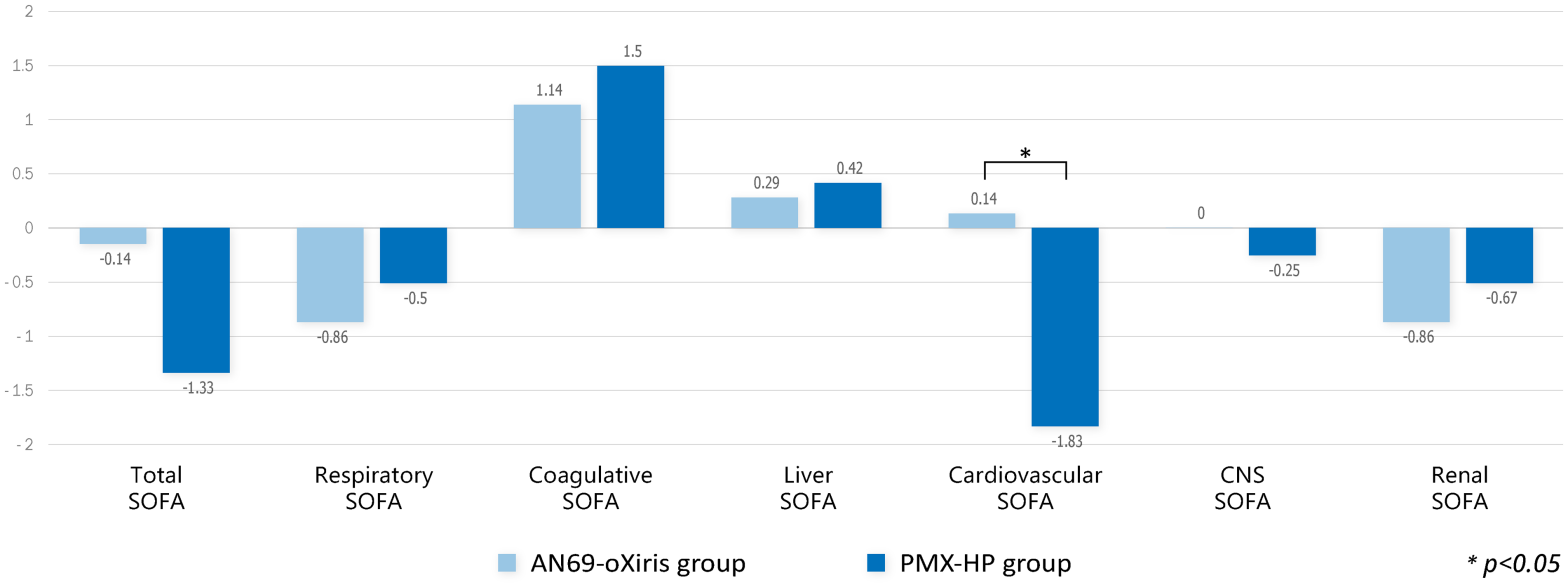
Comparison of changes in SOFA scores after blood purification with different filters (modalities). Change of each value = T1 – T0.

A comparison of the changes in laboratory values before and after treatment and between treatment methods is shown in Figure 4. PLT counts significantly decreased 24 h after treatment in both groups compared to baseline values, whereas no difference in Cr or lactate levels was seen after treatment. The mean pH value in the AN69-oXiris group increased significantly after treatment compared to the baseline value (*p* = 0.034). Figure 5 represents changes in SOFA scores before and after each treatment and a comparison of the results according to treatment modality. The AN69-oXiris group showed lower respiratory SOFA scores after 24 h of treatment than the PMX-HP group [0.5 (IQR = 0–1) vs. 2 (IQR = 1–3), respectively, *p* = 0.041]. However, no difference was observed in changes in respiratory SOFA scores before and after treatment between the two groups. The changes in cardiovascular SOFA scores at baseline and 48 h after treatment were significant in the PMX-HP group compared to the AN69-oXiris group [−1.5 (IQR = −4 – 0) vs. 0 (IQR = −1 – 1), respectively, *p* = 0.035].

The results of the comparative analysis of clinical outcomes are shown in Table 3. No difference was seen between the two groups in the incidence of postoperative complications or mortality. Regarding the recovery of kidney function after blood purification treatment, significantly more patients in the PMX-HP group had complete recovery than the AN69-oXiris group [12 (100%) vs. 2 (33.3%), respectively, *p* = 0.005]. The AN69-oXiris group showed significantly lower costs during ICU admission or hospitalization than the PMX-HP group ($17,727 ± 10,741 USD vs. $31,569 ± 19,345 USD, *p* = 0.049 and $27,007 ± 14,532 USD vs. $46,437 ± 29,276 USD, *p* = 0.048, respectively) despite similar lengths of ICU stays or hospitalization.

**Table 3:** Comparison of clinical outcomes between different filters.

Clinical outcomes	AN69-oXiris group	PMX-HP group	*p*-value
	*N* = 6 (33.3%)	*N* = 12 (66.7%)	
Recovery of kidney function after 28 days			
Complete recovery^a^	2 (33.3)	12 (100)	0.005
Partial recovery^b^	2 (33.3)	0	0.098
Dependence on dialysis^c^	2 (33.3)	0	0.098
Length of ICU stay, day (mean±SD)	7.33±4.32	9.16±5.83	0.507
Length of hospital stay, day (mean±SD)	24.83±10.52	32.92± 23.93	0.446
Total cost during ICU admission, $ (mean±SD)	17,727±10,741	31,569±19,345	0.049
Total cost during hospitalization, $ (mean±SD)	27,007±14,532	46,437±29,276	0.048
Postoperative complications	3 (50)	5 (41.7)	1.000
Anastomosis leakage	0	0	
Intraabdominal fluid collection	2 (33.3)	0	0.098
Pneumonia	1 (16.7)	6 (50)	0.316
Postoperative bleeding	0	0	
Wound infection	1 (16.7)	1 (8.3)	1.000
Postoperative ileus	1 (16.7)	3 (25)	1.000
Vascular complication	0	0	
Newly-onset arrhythmia	0	2 (16.7)	0.529
ICU mortality	0	3 (25)	0.515
7-day mortality	0	2 (16.7)	0.529
28-day mortality	0	3 (25)	0.515
In-hospital mortality	0	3 (25)	0.515

SD, standard deviation; IQR, interquartile range.

a Complete recovery was defined as a recovery to normal serum Cr and normal urine analysis after 28 days of treatment initiation.

b Partial recovery was defined as no return to normal serum Cr or urine analysis after 28 days of treatment initiation, but no need for hemodialysis.

c Dependence on dialysis was defined as the keeping of hemodialysis after 28 days of treatment initiation.

**Figure 6:**
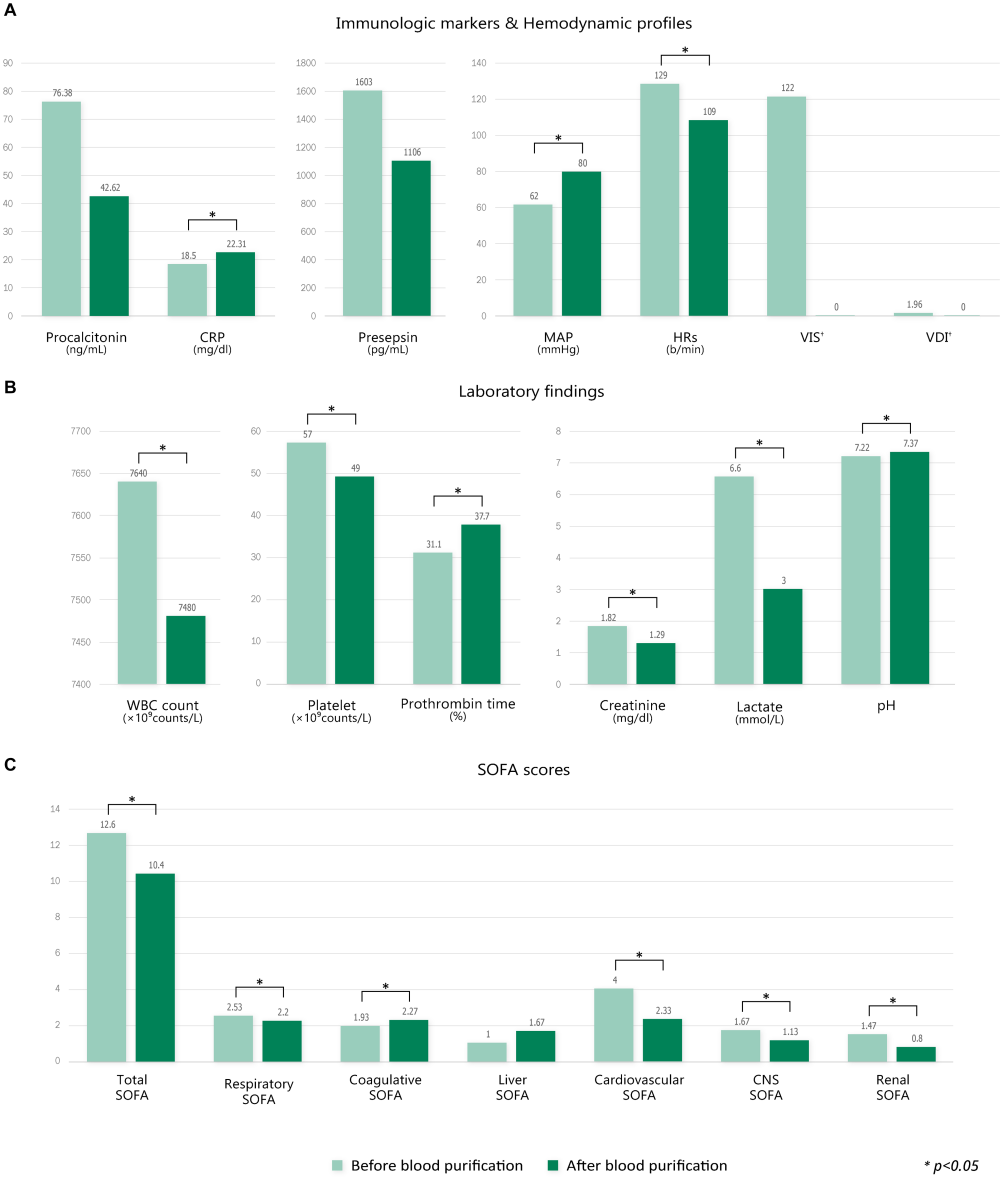
Comparison of changes in variable markers and profiles before and after blood purification in the PMX-HP group with high SOFA scores (SOFA scores ≥9). **(A)** Immunologic markers and hemodynamic profiles, **(B)** laboratory findings, and **(C)** SOFA scores. Change of each value = T1 – T0 (^†^Change of VIS, VDI = T2 – T0).

**Figure 7:**
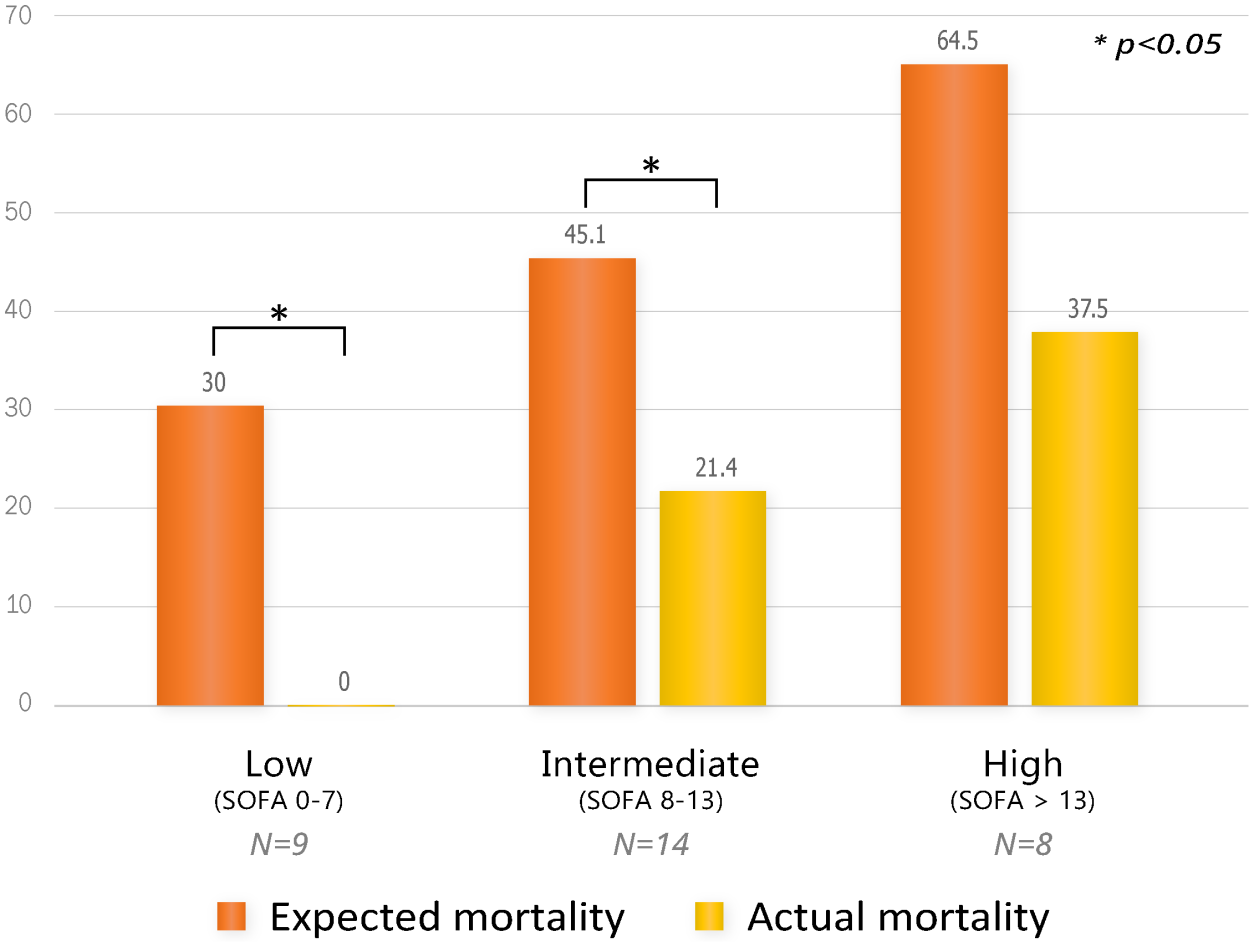
Comparison of mortality by subgroup according to SOFA scores in all patients who underwent PMX-HP treatment. Expected mortality was based on the APACHE II score, and actual mortality was based on 7-day mortality.

Since this study only analyzed patients in the PMX-HP group with disease profiles similar to patients in the AN69-oXiris group through PS-matching, PMX-HP patients with high disease severity were not included in the analysis. Therefore, we additionally performed a subgroup analysis on patients with high disease severity to assess the impact of PMX-HP treatment on more critically ill conditions. Figure 6 shows the changes in variable markers and profiles before and after PMX-HP treatments in patients with SOFA scores of 9 or higher. Laboratory parameters, as well as hemodynamic profiles, showed significant improvements after 24 h of PMX-HP treatment. Total SOFA scores and individual SOFA scores were significantly improved, except for liver SOFA scores. Figure 7 shows a comparison of mortality in all patients who underwent PMX-HP treatment. We calculated the expected mortality rate based on the APACHE II score, and the actual mortality rate was based on the 7-day mortality rate. Patients were divided into low disease severity groups (SOFA score 0–7), intermediate group (SOFA score 8–13), and high group (SOFA score > 13). Of the total 31 patients, 9 were in the low group, 14 were in the intermediate group, and 8 were in the high group, and the actual mortality rates were significantly lower than the expected mortality rates in the low and intermediate disease severity subgroups.

## Discussion

Our results showed that the hemodynamic status, reflected by VIS, HR, and MAP of patients with abdominal septic shock, improved after blood purification treatment. A significant decrease in cardiovascular SOFA scores was seen in the PMX-HP group, but no difference was observed in postoperative complication rates or mortality between the two groups. Substantial enhancements in hemodynamic status and SOFA scores were seen in the subgroup analysis of the PMX-HP group with higher disease severity, as well as significantly reduced mortality.

The excessive release of vasoactive substances or cytokines in septic shock results from host immune responses against endotoxins and leads to a decrease in systemic vascular resistance, cardiac contractility, and compensatory increases in heart rate (24). When the host’s macrophages are activated in response to endotoxin, an endogenous cannabinoid, such as anandamide, is produced, which subsequently triggers vasodilation that ultimately leads to hypotension (25). Blood purification treatments, such as PMX-HP or AN69-oXiris, can remove endotoxins and excess cytokines to improve systemic dysregulation and hemodynamic instability. Garcia-Ramos et al. showed that PMX-HP treatment reduced mortality in postoperative patients with abdominal septic shock to 25%, significantly lower than the 57.98% predicted by APACHE II scores (26). Zang et al. also reported that using AN69-oXiris for septic patients in the surgical ICU was associated with improved hemodynamic status and decreased cytokine levels (5). In this study, both groups showed increased MAP, decreased HR, and reduced need for vasopressors, as well as improved lactate and pH levels after blood purification treatment. We supposed that the appropriate application of blood purification treatment in addition to standard resuscitation will have clinical benefits in selected patients, especially those with refractory septic shock that persists even after the complete elimination of the infectious focus.

In this study, we used two different types of blood purification modalities, PMX-HP and AN69-oXiris. PMX-HP consists of a cartridge containing polystyrene-derived woven fibers with polymyxin B immobilized on the surface. Polymyxin B is an antibiotic that works by directly binding to lipopolysaccharide on the outer cell wall of GNB, allowing PMX-HP to specifically remove endotoxin (14, 27, 28). Our results showed that the PMX-HP group had a more prominent reduction in VISs and improvement in cardiovascular SOFA scores compared to the AN69-oXiris group. These findings might be attributed to differences in the endotoxin removal capacity between the filters of the two modalities. In an *in vitro* study by Malard et al. comparing the ability to adsorb inflammatory mediators, PMX-HP showed a significantly higher endotoxin clearance rate than AN69-oXiris in the first 30 min (14). In another *in vitro* study, AN69-oXiris cleared endotoxin at a rate of 10% after 4 h, whereas PMX-HP still had a clearance rate of 70% after 4 h (15). The device adsorption capacity, which refers to the amount of endotoxin a device can eliminate from whole blood, is 64 μg for PMX-HP compared to 1–8 μg for AN69-oXiris (29). These support the hypothesis that PMX-HP may be more favorable in cases of high endotoxin levels due to its ability to remove large amounts of endotoxin at a faster rate. The rapid clearance of endotoxin allows for the early resolution of refractory shock symptoms, such as hypotension, and faster reductions in the vasopressor dosage to minimize the risk of vasopressor complications related to poor outcomes (30). Despite some conflicting results in previous studies, a post-hoc analysis of the EUPHRATES trial reported a significant reduction in mortality when PMX-HP was applied to patients with moderate-to-severe endotoxemia defined as an endotoxin activity assay (EAA) level of 0.6 to 0.9 (8, 13). Our previous study also showed that the application of postoperative PMX-HP could significantly improve in-hospital mortality in patients with moderate or greater endotoxemia, defined as an EAA level of 0.54 or greater (31). In current study, we demonstrated similar results in subgroup analysis of patients with high SOFA score of 9 or higher, the PMX-HP group improved hemodynamic status such as MAP or HRs. Interestingly, in the comparison of mortality, the 7-day mortality rate was significantly lower than predicted mortality not only in the low disease severity group (SOFA scores 0–7) but also in the intermediate disease severity group (SOFA scores 8–13). Hence, PMX-HP could be a feasible option, especially in patients with moderate-to-severe disease severity as defined by endotoxin levels. Further prospectively designed research is needed to establish objective criteria for selecting PMX-HP over AN69-oXiris.

Another blood purification modality, AN69-oXiris treatment, has a unique filter structure with a three-layer membrane. The first layer is an AN69 copolymer hydrogel structure that facilitates the adsorption of cytokines, such as interleukin (IL)-6, IL-8, tumor necrosis factor (TNF)-α, and interferon (IFN)-γ. The second layer is multiple layers of polyethyleneimine (PEI), which is highly positively charged and can adsorb endotoxins, and the third layer is coated with heparin to reduce local thrombogenicity (32). In addition to immunomodulatory support, this treatment can provide renal support with acid–base and electrolyte correction and volume control by CRRT0. The serum Cr levels of all patients in the AN69-oXiris group decreased 24 h after treatment, and two patients had a complete recovery of renal function after 28 days. Thus, AN69-oXiris may be a favorable option for patients with septic shock, where the incidence of AKI is up to 50%. However, the obvious effect on mortality is uncertain, and it might not be as effective as PMX-HP in removing endotoxins. Additionally, AN69-oXiris can adsorb antibiotics, such as vancomycin and amikacin, with a risk of lowering the circulating levels of administered antibiotics below therapeutic concentrations (32). Thus, the use of AN69-oXiris can be limited in patients with high endotoxin levels or high disease severity. Nevertheless, our results showed that AN69-oXiris was a more cost-effective option for blood purification treatment than PMX-HP. The cost of filters differs between countries. However, the implementation of the AN69-oXiris is typically less expensive than that of PMX-HP. In South Korea, the cost of the PMX-HP filter is approximately 3.2 times as much as that of AN69-oXiris, about twice as expensive in Taiwan, and roughly 2.25 to 3 times as expensive in European countries. While AN69-oXiris includes a CRRT function, PMX-HP requires an additional CRRT machine for renal replacement therapy, contributing to its higher cost compared to AN69-oXiris. Thus, AN69-oXiris can be a more beneficial choice for patients with sepsis or septic shock with relatively mild endotoxemia, as it offers comparable clinical outcomes at a lower cost. While various blood purification modalities have been developed, clinical comparisons between them have not yet been studied, and SSC guidelines do not yet recommend the use of blood purification treatment due to the inconsistent results of the clinical efficacy of each modality (18, 19). Thus, It is necessary to understand the capabilities and characteristics of each modality and provide a treatment strategy based on patient conditions, such as endotoxin levels, renal function, or disease severity, through well-designed prospective studies with larger populations in the near future.

Despite these interesting results, the study had inevitable limitations. First, selection bias may have been present due to the retrospective non-randomized design, and some details of the treatment protocols could be heterogeneous. Second, the PMX-HP group also underwent additional CRRT as needed. The study showed that more patients achieved complete recovery of kidney function in the PMX-HP group compared to the AN69-oXiris group. This appears to be the effect of applying additional CRRT in patients in the PMX-HP group, which offsets the impact of the absence of renal support function, a limitation of conventional PMX-HP therapy, and the patient’s kidney function is restored early as the CRRT function is added to the endotoxin removal effect due to the PMX-HP filter. However, due to this additional CRRT to the PMX-HP group, we were unable to confirm the clinical benefits of AN69-oXiris with CRRT function. In order to confirm the benefits of AN69-oXiris treatment in terms of preserving renal function, the next study will be a well-designed prospective study comparing patients treated with AN69-oXiris to patients treated with PMX-HP alone without CRRT. Also, as some patients in the PMX-HP group received CRRT with the AN69-ST filter, it is possible that the resulting cytokine clearance effect may have contributed to the reduction in VIS. To confirm this effect, we divided the PMX-HP group into 18 patients who received CRRT and 13 patients who did not, and found no significant difference in the changes in VIS. Due to the small number of cases, it is not possible to completely rule out the influence of the cytokine clearance effect of the AN69-ST filter based on this result alone. However, a previous study showed that although the reduction in VIS was observed in the AN69-ST group, the reduction was significantly greater in the AN69-oXiris group compared to the AN69-ST group (5). In our study, results show that the PMX-HP group had a significantly greater reduction in VIS compared to the AN69-oXiris group. This could be due to the effect of PMX-HP rather than the AN69-ST filter in CRRT machine concurrently used with PMX-HP. Third, because the measurement of EAA levels was only available for a subset of participants, we were unable to stratify the effectiveness of modalities according to endotoxin levels or identify differences in outcomes between the two modalities. Additionally, the study was conducted on patients of a single race at a single center with a small sample size, which could potentially limit the applicability of our results to broader populations. Finally, because the analysis was performed after PS-matching, there was no difference in initial disease severity between the patient groups ultimately analyzed. This made it difficult to provide objective evidence for selecting a treatment modality based on disease severity. In addition, considering the characteristics of PMX-HP, which may be useful in relatively severe cases, a meaningful comparative analysis of the two modalities requires the inclusion of severe cases. We sub-analyzed the entire PMX-HP group according to disease severity. And we found a significant improvement in mortality after PMX-HP treatment not only in patients with mild septic condition, but also in severe patients. This finding supports our hypothesis that PMX-HP treatment could be a better option for moderate-to-severe endotoxemic patients with severe clinical deterioration. The limited number of cases in each group restricts the conclusions. However, to the best of our knowledge, this was the first clinical trial to compare and analyze two different blood purification modalities, as many previous studies examined each modality separately. Although this study included a small number of subjects, the authors believe that the results comparing the efficacy of the two modalities according to disease severity will be clinically meaningful. Considering that the high cost of these treatments is a major obstacle to their implementation, the results of this study may provide clues to the actual clinical differences between two treatments. In this way, it would be useful to compare different blood purification methods in critically ill patients before conducting large-scale prospective randomized controlled trials. Additionally, we selectively applied blood purification treatments to patients with abdominal septic shock whose infectious sources were successfully controlled by surgery. The authors believe that such patient characteristics could block the consistent inflow of endotoxin, making blood purification treatment more clinically efficient. We believe that the clinical implication should be different in non-successfully source-controlled patients or medical sepsis populations, and the use of hemoperfusion modalities would be more effective in patients with refractory shock with abdominal septic origins. Further studies should be conducted to overcome several limitations, and a subgroup analysis stratified by endotoxin levels should be performed to identify the therapeutic range of efficacy for each modality and provide tailored indications.

## Conclusion

In conclusion, PMX-HP and AN69-oXiris are potential therapeutic options for patients with refractory septic shock with intra-abdominal origins, especially after the surgical elimination of the infectious source. Further large-scale, prospective, randomized controlled trials that take into account patient characteristics, such as disease severity or cost burden, are needed to provide detailed guidance for blood purification treatment.

## Data availability statement

The raw data supporting the conclusions of this article will be made available by the authors, without undue reservation.

## Ethics statement

The studies involving humans were approved by Institutional Review Board of the Catholic University of Korea. The studies were conducted in accordance with the local legislation and institutional requirements. The participants provided their written informed consent to participate in this study.

## Author contributions

HK: Conceptualization, Data curation, Formal analysis, Investigation, Methodology, Visualization, Writing – original draft, Writing – review & editing. YC: Data curation, Investigation, Writing – review & editing. GL: Data curation, Investigation, Writing – review & editing. EK: Conceptualization, Formal analysis, Methodology, Project administration, Supervision, Validation, Writing – review & editing.
